# Response to the letter to the editor regarding “Health economic evaluation of microprocessor and non-microprocessor-controlled prosthetic knees”

**DOI:** 10.33137/cpoj.v8i2.46486

**Published:** 2025-12-31

**Authors:** C.E. Bosman, C.K. van der Sluis, A.H. Vrieling, J.H.B. Geertzen, B.L. Seves, H. Groen

**Affiliations:** 1 Department of Rehabilitation Medicine, University of Groningen, University Medical Center Groningen, Groningen, The Netherlands.; 2 Department of Epidemiology, University of Groningen, University Medical Center Groningen, Groningen, The Netherlands.

**Keywords:** Lower Limb, Amputation, Prostheses, Cost Analysis, Quality of Life, Questionnaire, Mobility, Microprocessor Knee, Cost-effectiveness

Dear Editor,

We thank colleagues Brüggenjürgen, Riemer and Gapp for their detailed consideration and for recognizing the quality of our study.^1^

We appreciate that when comparing two lower-limb prosthetic devices across a range of prices, such as microprocessor-controlled and non-microprocessor-controlled prosthetic knees, the average reader would not expect an incremental cost-effectiveness ratio that exceeds values observed for expensive drugs such as monoclonal antibodies. However, it should be noted that the ratio not only reflects the difference in costs, but also the difference in effects. As the difference in effects becomes very small, the ratio will increase very sharply. In our case, the small QALY difference explains the high cost-effectiveness ratio, even though the cost difference is not excessive.

With respect to the costs of prosthesis, we would like to clarify the design of our study to avoid misunderstanding. It is true that we included participants who had their first prosthesis fitted, as well as those who received a replacement. However, we treated both groups as if they were new fittings, in our comparison of prosthesis types. This was a deliberate choice to ensure a consistent cross-sectional comparison of prosthesis types. Consequently, we assigned full costs of their respective prosthesis to all participants. We acknowledge that this approach influences ICUR estimates and agree that assumptions about replacement cycles warrant further investigation.

We regret that the impression was given that a very short replacement cycle would be required. In fact, in the first paragraph of our discussion, we refer to a previous study that suggests that cost-effectiveness of the MPK may improve in the long term due to lower healthcare costs related to falls. Thus, the higher initial costs of MPK could be at least partially compensated.

We appreciate your recalculations of ICUR,^2^ but we feel that the proposed corrections to our ICUR are based on assumptions beyond our dataset; they illustrate the sensitivity of ICUR to prosthesis life-cycle assumptions. The calculations show the importance of the parameters such as prosthesis life-cycle and stress the importance of future studies evaluating long-term outcomes. Also, development of a quality-of-life measurement instrument that is more sensitive to prosthesis-specific outcomes, to be translated into a utility value, would be a big step forward in evaluating the cost-effectiveness of prostheses.
